# An unusual case of colon vascularization by the inferior mesenteric artery

**DOI:** 10.1590/1677-5449.009315

**Published:** 2017

**Authors:** Serghei Covanțev, Natalia Mazuruc, Olga Belic

**Affiliations:** 1 Nicolae Testemițanu State University of Medicine and Pharmacy – SUMPh Nicolae Testemitanu, Department of Human Anatomy, Chișinau, Republic of Moldova.

**Keywords:** inferior mesenteric artery variation, accessory middle colic artery, accessory left colic artery, variação da artéria mesentérica inferior, artéria cólica média acessória, artéria cólica acessória esquerda

## Abstract

In this article we present a rare variant in which the large intestine was vascularized by the inferior mesenteric artery. It was encountered during macro and microscopic dissection of the cadaver of a 63-year-old woman at a university department of human anatomy. In this case, the ascending, transverse, descending, and sigmoid colon and rectum were vascularized by the inferior mesenteric artery, whereas the small intestine, cecum and appendix were supplied by the superior mesenteric artery.

## Resumo

Neste artigo apresentamos uma variação rara em que o intestino grosso era vascularizado pela artéria mesentérica inferior. A variação foi descoberta durante a dissecção macro e microscópia de um cadáver do sexo feminino, 63 anos de idade, em um departamento universitário de anatomia humana. Neste caso, o cólon ascendente, transverso, descendente e sigmoide e também o reto eram vascularizados pela artéria mesentérica inferior, ao passo que o intestino delgado, ceco e apêndice eram vascularizados pela artéria mesentéria superior.

## INTRODUCTION

The arterial supply of the internal organs is characterized by a high degree of variation in origin, trajectory, and branching patterns. These variations frequently cause difficulties even for experienced specialists during surgical and diagnostic procedures. Current data covers the classical (more frequently encountered) variants of artery branching, which are mainly described in this domain.

Abdominal surgery is founded on a profound understanding of the blood supply to the abdominal organs. The greater part of descriptions in the literature relate to different variations of vascularization due to anomalies of the superior mesenteric artery, whereas the inferior mesenteric artery even now remains largely under studied. Knowledge of existing aberrations is important for planning or conducting surgical and radiological procedures.

## CASE DESCRIPTION

During dissection of a complex of internal organs of the abdominal cavity obtained from a 63-year-old female cadaver, we identified a rare variant of vascularization of the large intestine. The inferior mesenteric artery had its origin 4 cm above the aortic bifurcation and then divided into two trunks.

The first trunk branched into the right colic artery and the middle colic artery, supplying the ascending colon and the right half of the transverse colon. The right colic artery divided into ascending and descending branches. The middle colic artery also divided into two vessels that anastomosed with the ascending branch of the right colic artery and with the branches of the accessory middle colic artery. An accessory left colic artery took origin from the middle colic artery, and vascularized the descending colon.

The second trunk of the inferior mesenteric artery divided into accessory middle colic artery and left colic artery, which vascularized the left half of the transverse colon and the descending colon. The sigmoid artery began from the left colic artery and then gave off two superior rectal arteries as its branches.

**Figure 1:**
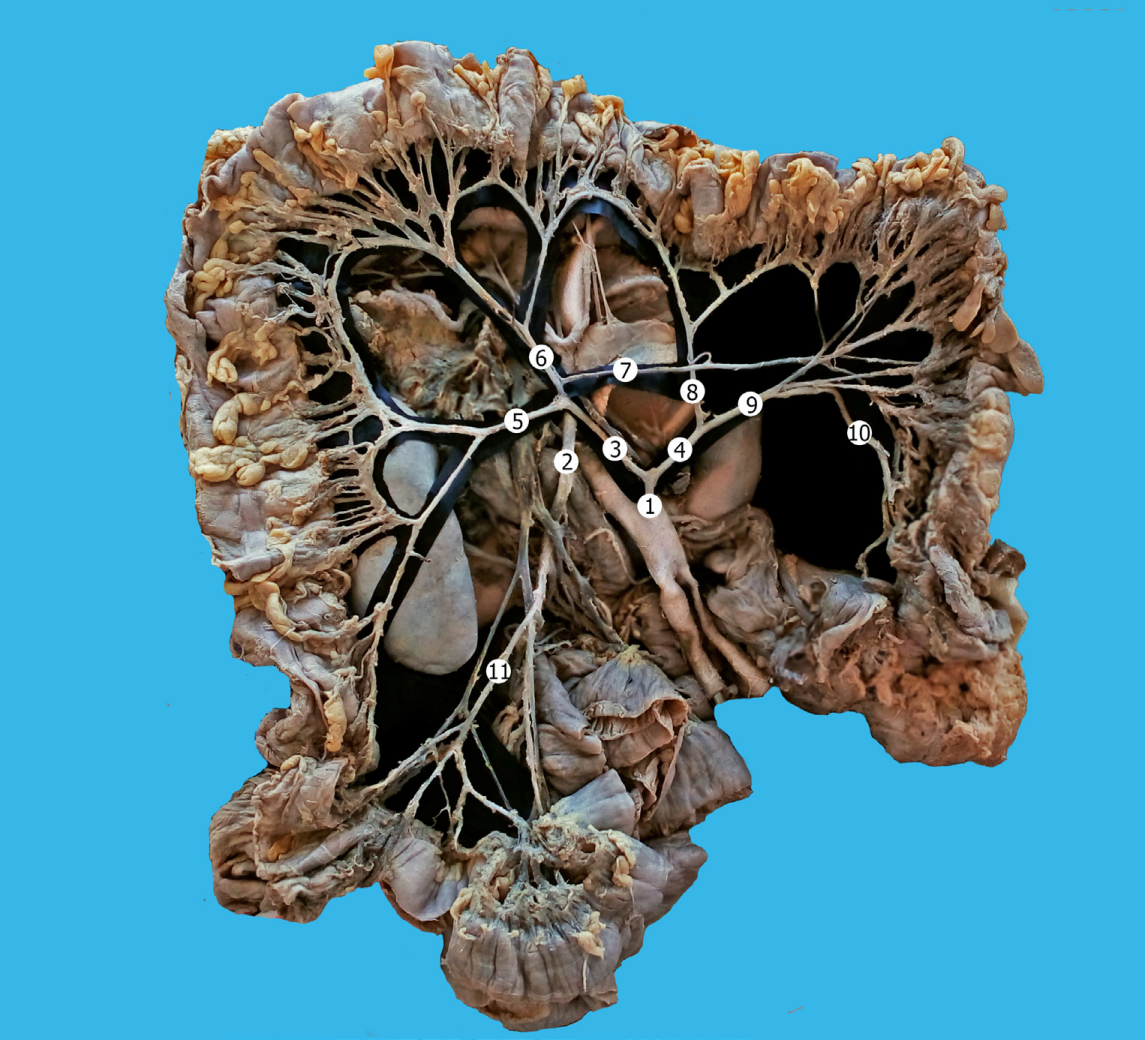
Vascularization of the large intestine. Macro specimen (female, 61 years). 1) inferior mesenteric artery; 2) superior mesenteric artery; 3) common trunk of the right colic and middle colic arteries; 4) common trunk of the accessory middle colic and left colic arteries; 5) right colic artery; 6) middle colic artery; 7) accessory right colic artery; 8) accessory middle colic artery; 9) left colic artery; 10) sigmoid artery; 11) ileocolic artery.

The small intestine, cecum, and appendix were vascularized by the superior mesenteric artery. There were no visible anatomical anastomoses between the superior and inferior mesenteric arteries (Figure 1).

## DISCUSSION

Current data indicate that the inferior mesenteric artery usually supplies the left 1/3 of the transverse colon, descending colon, sigmoid colon and rectum.

The arteries of the large intestine are characterized by a high degree of variability. In 4.28-23% of cases, the right colic artery and the middle colic artery begin together as a common trunk.^1^^,^ 2

The right colic artery is present in only 10-63% of cases, whereas the middle colic artery is present in 99.3% and in 7.2% there are accessory middle colic arteries.^3^^,^ 4

All the above-mentioned variations are characteristic for the superior mesenteric artery. Cases in which the inferior mesenteric artery is the main source of the blood supply to the large intestine are rarely found in the literature.

Wu et al.^5^ described a rare case in which the superior mesenteric artery was absent and the intestine was vascularized only by the inferior mesenteric artery. Based on their classification of superior–inferior mesenteric arterial variations, our case is a type I (the normal type) pattern, in which the superior mesenteric artery and the inferior mesenteric artery originate separately from the abdominal aorta. In our opinion it is also important to specify which parts of the intestine the artery supplies.

The arterial system of the gastrointestinal tract is originally segmental. It derives from a number of pairs of ventral splanchnic arteries. All of them have a segmental pattern and branch from the paired dorsal aorta. After fusion of the dorsal aortae, these arteries also fuse together forming unpaired trunks that provide arterial supply to the primitive digestive tube. The trunks are connected with each other by longitudinal anastomoses. Eventually, this system is simplified through reduction of the number of vessels, so only three of them remain: the celiac trunk and the superior and inferior mesenteric arteries.^6^ We presume that any changes in this process eventually cause the majority of vascular anomalies that can be found at this level, including our case.

The presence of accessory left colic arteries is also rarely described in the literature.^7^^,^ 8 The accessory left colic artery usually divides into two branches: the ascending and the descending, which form anastomoses with the middle and left colic arteries.^8^

The marginal artery of the colon may be absent at the splenic angle and so it is considered a zone at high risk of ischemia.^9^ This area is often called the Griffith point and is a zone where the anastomosis between the branches of superior and inferior mesenteric arteries can frequently be insufficient to maintain proper vascularization in cases of ischemia. An angiographic study conducted by Meyers showed that the anastomosis at the Griffith point is present in 48% of cases, weak in 9% of cases, and absent in 43% of cases.^10^ In our case, the marginal artery was absent in the region of the cecum between the right colic artery and the ileocolic artery. This type of variation can be encountered in 5% of cases and has been described by Steward and Rankin.^11^ We should underscore once again that in our case the ileocolic artery is a branch of the superior mesenteric artery, whereas the right colic artery is a branch of the inferior mesenteric artery making this type of variation even rarer.
